# Point prevalence mapping reveals hotspot for onchocerciasis transmission in the Ndikinimeki Health District, Centre Region, Cameroon

**DOI:** 10.1186/s13071-020-04387-6

**Published:** 2020-10-16

**Authors:** René Afor Aza’ah, Laurentine Sumo, Ngum Helen Ntonifor, Jean Bopda, Rolph H. Bamou, Hugues C. Nana-Djeunga

**Affiliations:** 1 Department of Biological Sciences, Faculty of Science, University of Bamenda, Bambili, Cameroon; 2 Centre for Research on Filariasis and other Tropical Diseases (CRFilMT), Yaounde, Cameroon

**Keywords:** Onchocerciasis, CDTI, Hotspot, Ndikinimeki Health District, Cameroon

## Abstract

**Background:**

Ivermectin-based preventive chemotherapy (PC) is distributed annually to all at-risk populations eligible for ivermectin treatment to control and/or eliminate onchocerciasis. Information on the impact of mass ivermectin administration on onchocerciasis transmission is scanty, and it is tricky to appreciate the progress towards elimination and engage corrective measures. To fill that gap in the Centre Region in Cameroon, the current onchocerciasis endemicity level in the Ndikinimeki Health District after about two decades of mass treatments was assessed.

**Methods:**

A cluster-based cross-sectional survey was carried out in the Ndikinimeki Health District and all volunteers aged ≥ 5 years were (i) interviewed on their compliance to ivermectin over the past five years and (ii) underwent clinical (nodule palpation and visual search for onchocercal lesions) and parasitological examinations (skin snip) for onchocerciasis.

**Results:**

The overall *Onchocerca volvulus* prevalence was 7.0% (95% CI: 5.2–9.3%). The prevalence of the disease was significantly higher in the communities Kiboum 1 and Kiboum 2 compared to the other communities (highest prevalence in Makénéné Town Water: 8.5%; 95% CI: 2.3–20.4%) (*χ*^2^ = 51.314, *df* = 11, *P* = 0.0001). The proportion of systematic non-compliers to ivermectin was 23.3% (95% CI: 19.9–27.1%) among individuals interviewed. In the sentinel sites (Kiboum communities), onchocerciasis prevalence decreased from 95.2% (95% CI: 88.3–98.1%) to 23.7% (95% CI: 14.7–36.0%).

**Conclusions:**

This study has revealed that the Ndikinimeki Health District is hypo-endemic for onchocerciasis after about two decades of preventive chemotherapy. However, transmission is ongoing, with potential hotspots in the Kiboum 1 and Kiboum 2 communities, which are known as first-line communities (closest to the breeding sites of the vector). Alternative or complementary strategies to annual ivermectin appear compulsory to accelerate the momentum towards onchocerciasis elimination.
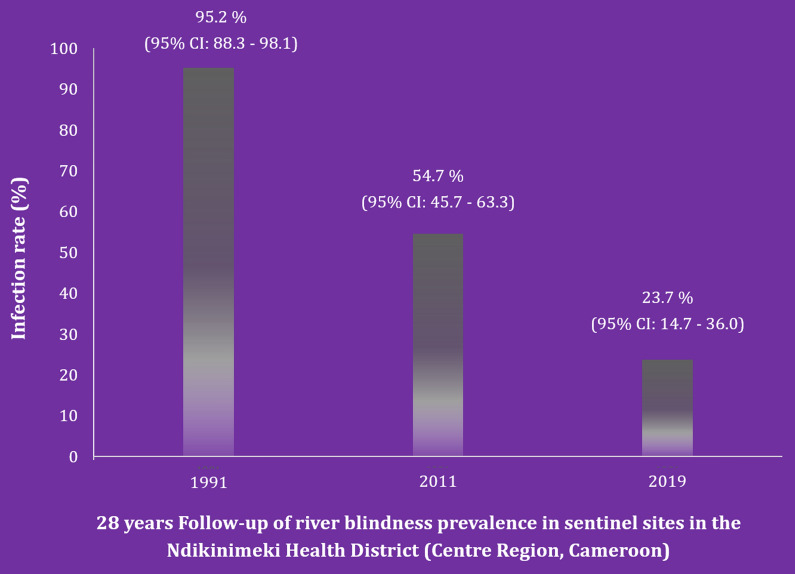

## Background

Onchocerciasis, better known as river blindness because of the high prevalence of blindness in villages located along fast-flowing rivers where the vectors breed [1], is a neglected tropical disease (NTD) affecting over 20.9 million people worldwide, of which 99% reside in Africa [2]. In Cameroon, onchocerciasis is endemic in all the ten regions with approximately six million people infected and about 60% of the population living in high risk areas for the disease [3]. This disease is caused by the parasitic nematode *Onchocerca volvulus* and transmitted *via* the bites of female blackflies of the genus *Simulium* during blood-feeding. People at highest risk of acquiring onchocerciasis are those living or working near fast-flowing and well-oxygenated streams or rivers appropriate to blackfly breeding. This infection rarely causes death but imposes suffering, stigmatization, and hardship to affected individuals and communities. It is the second leading cause of infectious blindness worldwide after trachoma [4]. The pathogenesis of the diseases is essentially due to microfilariae, while adult worms usually induce no pathology or simply stimulate the development of characteristic subcutaneous nodules.

Ivermectin (Mectizan®) is the only known effective and safe drug used for preventive chemotherapy (PC) against onchocerciasis [5]. Since this drug has a limited macrofilaricidal activity [6] and adult *O. volvulus* is long-lived (lifespan > 14 years [7]), treatments must be repeated yearly for 12–15 years to break the transmission cycle [8]. Community-directed treatment with ivermectin (CDTI), formally adopted by the African Programme for Onchocerciasis Control (APOC) in 1997, has so far remained the main control strategy to eliminate this disease in Africa. Indeed, CDTI relies on active community participation, by focusing on empowering communities to take responsibility for ivermectin delivery, deciding how, when and by whom the ivermectin treatment should be administered [2]. This strategy, implemented once-a-year in most African countries, including Cameroon, has significantly improved ivermectin treatment coverage [9, 10]. Unlike Latin America where the bi- or multi-annual large-scale treatment with ivermectin has succeeded in eliminating the infection in four of the six endemic countries (Columbia in 2013, Ecuador in 2014, Mexico in 2015 and Guatemala in 2016) [11], transmission of the disease has been interrupted only in limited foci in some endemic countries in Africa (Mali, Senegal and Nigeria) [12–14]. In most foci in Africa, including in Cameroon, the disease is persisting with prevalence up to 50% despite almost three decades of CDTI [15–18]. In order to improve current control approaches and define a more pinpointed strategy, it is worth carrying out regular surveys to assess the impact of control approaches on the disease epidemiology. Therefore, this study assessed onchocerciasis endemicity in the Ndikinimeki Health District after about two decades of CDTI, using a district-wide rather than sentinel site approach.

## Methods

### Study area

The study was carried out in the Ndikinimeki Health District, chosen because onchocerciasis-related monitoring and evaluation information was scanty or only sentinel site-based, although ivermectin mass administration was ongoing since 1998. The Health District consists of six Health Areas namely Makenene, Nitoukou, Nyokon, Ndokowanen, Ndikinimeki and Boutourou. The Ndikinimeki Health District (4.76°N, 10.83°E) is located in the Mbam and Inoubou Division of the Centre Region of Cameroon. The climate is equatorial and divided into four seasons: a long dry season (November-February), a short rainy season (March-June), a short dry season (June-August) and a long rainy season (August-November) [19]. The mean annual temperature is 22.4 °C and the average annual rainfall is 1440 mm. The region is highly irrigated by the rivers Inoubou, Makombé and Makénéné and their various tributaries. The main activities of the inhabitants are agriculture (cocoa farming and fishing) and sand mining. The Health District has a population size of about 44,519 inhabitants based on the 2017 health population denominators [20].

### Study design and participants

A cross-sectional survey was carried out in the Ndikinimeki Health District following a cluster sampling approach. Clusters were selected using the probability proportionate to estimate size (PPES) strategy. In order to ensure that all clusters have the same probability of selection, communities (clusters) were organized into Health Areas prior to the PPES procedure to take into account the difference in the number of communities per Health Area and the difference in community size. A total of 12 clusters were selected by the PPES procedure. Considering the onchocerciasis prevalence (54.7%) in the Ndikinimeki Health District according to the most recent impact assessment survey (2011) by the National Onchocerciasis Control Programme (NOCP), a minimal sample size of 381 was needed to estimate the true prevalence of onchocerciasis in 2019 in the Ndikinimeki Health District with 5% precision, and 95% confidence interval. Eligible participants were both males and females, aged 5 years and above, and who had been living in the selected clusters for at least 5 years. In each cluster, socio-demographic data, history of, and adherence to ivermectin treatments during the past 5 years preceding the survey, were collected from eligible participants using a structured questionnaire. Although eligible to ivermectin-based PC, individuals aged < 10 years were excluded from history and adherence to CDTI since they are less likely to provide accurate enough answers to such questions. All the individuals who accepted to participate underwent clinical and parasitological examinations, respectively. Furthermore, follow-up of the trend in onchocerciasis prevalence over three decades (or 25 years of annual mass ivermectin treatment) was carried out only in the Boutourou Health Area where a sentinel site (Kiboum communities) was identified during mapping exercise, based on the availability of baseline data. Both the baseline (1991) and the follow-up (2011) surveys were carried out by the NOCP, with the support of APOC (NOCP, unpublished reports).

### Clinical examination

The clinical examination included nodule palpation, visual inspection for skin disease and anamnesis for pruritus. Nodule palpation was performed on partially disrobed participants in a closed but well-illuminated room. Attention was paid to bony prominences of the torso, iliac crests, and upper trochanter of the femurs as was already described as election sites of onchocercal nodules in Africa [21]. Participants were also asked whether they suffer from pruritus and their skin visually inspected for cutaneous signs of onchocerciasis, especially depigmentation (leopard skin) and skin rashes [21, 22].

### Parasitological examination

Two skin snips were taken with a sterile 2 mm corneoscleral punch (type Holth) from the two posterior iliac crests of each participant. The skin snips were placed in two separate wells of a microtitration plate containing ~ 200 µl of normal saline (0.9%) and incubated for 24 h at room temperature. The fluid of each well of the microtitration plate was examined for *Onchocerca* microfilariae using bright-field microscopy under low magnification (40×) [23]. The number of microfilariae in the fluid was counted (when positive) and the individual microfilarial densities were computed as the arithmetic mean number of microfilariae in the two skin snips (mf/ss).

### Statistical analyses and onchocerciasis distribution map

All data collected as part of this study (clinical signs, nodules, mf count and compliance with ivermectin PC) were recorded into a purpose-built Microsoft Access database and subsequently exported into a Predictive Analytics Software (PASW) Statistics version 18 (SPSS Inc., Chicago, IL, USA) for statistical analyses. Prevalence of skin microfilariae, skin disease and onchocercal nodules (whatever the number of nodules per participant) were expressed as the percentage of infected or affected individuals among the total number of individuals examined. Compliance with ivermectin PC was defined as the proportion of individuals eligible to ivermectin who actually ingested the drug. The 95% confidence interval (CI) for proportion/prevalence was calculated using the Wilson method not corrected for continuity [24]. The intensity of infection was evaluated using two metrics, the skin microfilarial density and the community microfilarial load (CMFL). The skin microfilarial density was defined as the arithmetic mean of individual microfilarial densities with standard deviation (SD) to account for sampling fluctuations. The CMFL was computed as the geometric mean number of microfilariae per skin snip (mf/ss) among adults aged 20 years or more, using the log(x+1)-transformation to take into account zero counts [25], especially after multiple rounds of ivermectin treatments. Chi-square, Mann-Whitney and Kruskal–Wallis tests were used to compare onchocerciasis prevalence and mean microfilarial density between clusters, gender and age groups, respectively.

The geographical coordinates of each community visited were recorded using a high sensitivity global positioning system [GPS eTrex; Garmin (Europe) Ltd, Southampton, UK]. A thematic analysis was performed using a geographical information system (GIS) software (ArcGIS, version 10.2; ESRI Inc., Redlands, CA, USA) to map the distribution, at cluster (community) level, of onchocerciasis point prevalence in the Ndikinimeki Health District; three main layers (roads, vegetation cover and rivers/streams, the latest representing potential blackfly breeding sites) were used to create/illustrate the map.

## Results

A total of 603 participants aged 5 to 88 (median: 32; interquartile range (IQR): 42) years were examined in 12 clusters (communities) of the six Health Areas of the Ndikinimeki Health District. The sex ratio (M/F) was 1.04 (proportion of males: 50.9%).

### Prevalence and intensity of *O. volvulus* infection

**Fig. 1:**
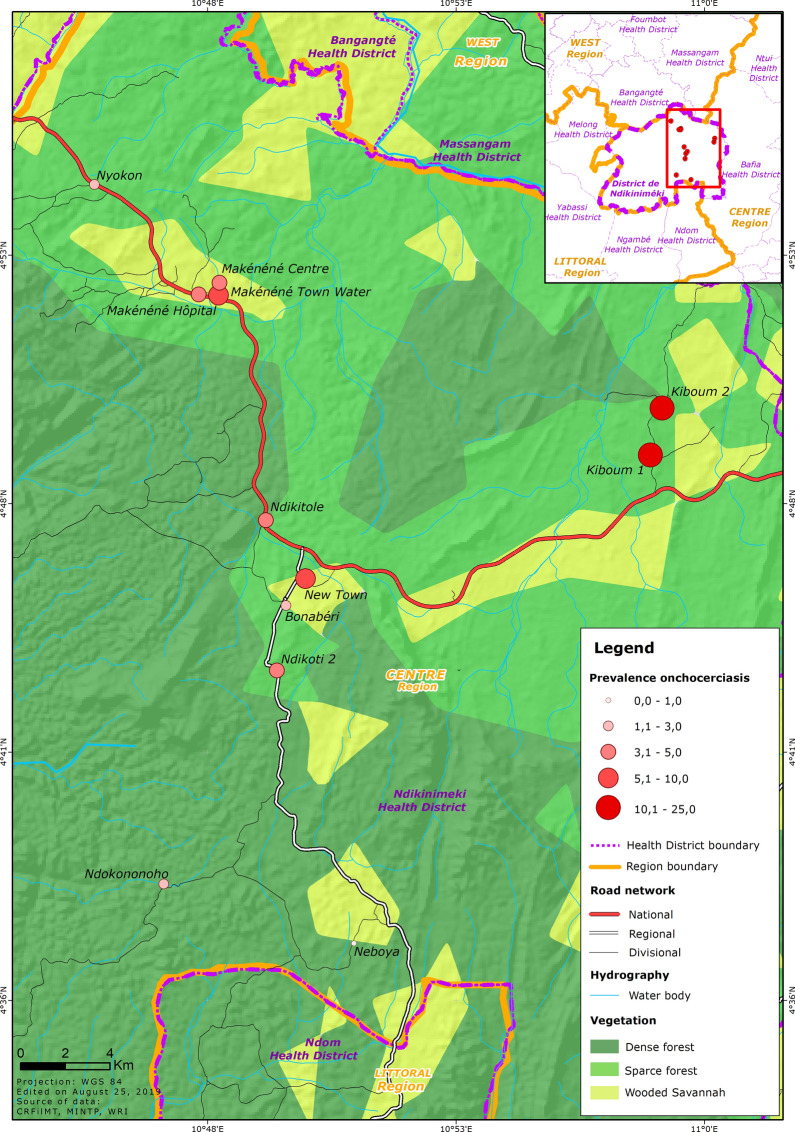
*Onchocerca volvulus* infection rate in the different communities surveyed in the Ndikinimeki Health District

Of the 603 participants examined, 42 (7.0%; 95% CI: 5.2–9.3%) were found to be infected with *O. volvulus*. The prevalence of onchocerciasis was significantly higher in the Boutourou Health Area (21.6% (95% CI: 14.5–30.1%) (*χ*^2^ = 48.708, *df* = 5, *P* = 0.0001), among males (11.1%; 95% CI: 7.7–15.1%) (*χ*^2^ = 16.301, *df* = 1, *P* = 0.0001) and among younger adults (20–34 years-old) (18.3%; 95% CI: 11.0–27.6%) (*χ*^2^ = 25.727, *df* = 3, *P* = 0.0001) (Table 1). At the community or cluster level, onchocerciasis prevalence ranged between 0% (95% CI: 0–8.8%) and 23.7% (95% CI: 13.6–36.6%). A significantly higher burden of the disease was observed in the communities Kiboum 1 (19.3%; 95% CI: 10.0–31.9%) and Kiboum 2 (23.7%; 95% CI: 13.6–36.6%) compared to the other communities (highest prevalence in Makénéné Town Water: 8.5%; 95% CI: 2.3–20.4%) (*P* = 0.0001) (Fig. 1, Table 2) (Additional file 1: Table S1). Furthermore, a significantly higher prevalence of the disease was observed among farmers (*P* = 0.0001).

**Table 1:** Prevalence of onchocerciasis in the Ndikinimeki Health District according to health areas, gender and age

Variable	No. of individuals examined	No. of individuals infected	Prevalence (%) (95% CI)
Health area			
Boutourou	116	25	21.6 (14.5–30.1)
Makénéné	160	8	5.0 (2.1–9.6)
Ndikinimeki	198	7	3.5 (1.4–7.1)
Ndokowonen	37	1	2.7 (0–14.2)
Nitoukou	40	0	0.0 (0–8.8)
Nyokon	52	1	1.9 (0–10.3)
Gender group			
Males	307	34	11.1 (7.7–15.1)
Females	296	8	2.7 (1.2–5.3)
Age group (years)			
5–19	217	5	2.3 (0.7–5.3)
20–34	93	17	18.3 (11.0–27.6)
35–49	109	8	7.3 (3.2–14.0)
≥ 50	184	12	6.5 (3.4–11.1)
Overall	603	42	7.0 (5.2–9.3)

*Abbreviation*: CI, confidence interval

**Table 2:** Intensity of *Onchocerca volvulus* infection in the Ndikinimeki Health District according to health area, gender and age

Variable	No. of individuals examined	Microfilarial density (mf/ss)		CMFL (mf/ss)
		Mean ± SD	Range	
Health area				
Boutourou	116	1.608 ± 6.939	0–63.5	0.465
Makénéné	160	0.641 ± 4.002	0–37.5	0.165
Ndikinimeki	198	0.189 ± 1.189	0–12.0	0.103
Ndokowonen	37	0.230 ± 1.397	0–8.5	0.084
Nitoukou	40	0	0	0
Nyokon	52	0.019 ± 0.139	0–1.0	0.021
Gender group				
Males	307	0.746 ± 3.584	0.0–37.5	0.266
Females	296	0.361 ± 3.978	0–63.5	0.079
Age group (years)				
5–19	217	0.168 ± 1.804	0–25.5	–
20–34	93	1.785 ± 7.908	0–63.5	0.357
35–49	109	0.321 ± 1.924	0–18.0	0.096
≥ 50	184	0.535 ± 2.945	0–27.5	0.125
Overall	603	0.557 ± 3.784	0–63.5	0.168

*Abbreviations*: mf/ss, microfilariae/skin snip; CMFL, community microfilarial load

The overall microfilarial density was 0.557 ± 3.784 (mean ± SD) mf/ss, significantly higher in the Boutourou Health Area (*χ*^2^ = 47.576, *df* = 5, *P* = 0.0001), among males (Mann Whitney U-test: *U* = 41645.5, *Z* = − 4.016, *P* = 0.0001) and young adults aged 20–34 years (*χ*^2^ = 25.703, *df* = 3, *P* = 0.0001) (Table 2). The intensity of infection was also significantly higher in Kiboum 1 (1.395 ± 8.414) and Kiboum 2 (1.814 ± 5.199) communities compared to other communities (highest microfilarial density in Makénéné Town Water: 0.979 ± 4.264) (*χ*^2^ = 50.490, *df* = 11, *P* = 0.0001). The overall CMFL in the study area was 0.168 mf/ss, significantly higher in the Kiboum 1 (0.383 mf/ss) and Kiboum 2 (0.538 mf/ss) communities (Additional file 1: Table S1).

### Morbidity associated with onchocerciasis

The overall prevalence of palpable nodules was 0.3% (95% CI: 0.1–1.2%), ranging between 0–0.6% but the difference was not significant between Health Areas (*χ*^2^ = 1.416, *df* = 5, *P* = 0.923), communities (*χ*^2^ = 8.768, *df* = 11, *P* = 0.643), age groups (*χ*^2^ = 2.892, *df* = 3, *P* = 0.409) and gender groups (*χ*^2^ = 0.001, *df* = 1, *P* = 1).

The overall prevalence of skin depigmentation and rashes was 3.0% and 0.3%, respectively, and no significant difference (*P* > 0.330) was found between Health Areas (*χ*^*2*^ = 5.762, *df* = 5, *P* = 0.330). The difference was also not significant between gender groups (*χ*^2^ = 0.154, *df* = 1, *P* = 0.694), age groups (*χ*^2^ = 1.037, *df* = 3, *P* = 0.792) and clusters (*χ*^2^ = 9.015, *df* = 11, *P* = 0.620).

Regarding pruritus, the proportion of individuals affected was significantly higher in the Nyokon Health Area (26.9%; 95% CI: 16.8–40.3%) compared to the other Health Areas (*χ*^2^ = 21.925, *df* = 5, *P* = 0.001), the difference was also significant when considering clusters (*χ*^2^ = 92.731, *df* = 11, *P* < 0.0001) and age groups (*χ*^2^ = 18.498, *df* = 3, *P* < 0.0001), but the difference was not significant when considering gender (*χ*^2^ = 1.436, *df* = 1, *P* = 0.231).

### History and adherence to ivermectin treatment

Overall, 74.8% (95% CI: 71.2–78.1%) of participants reported that they have swallowed ivermectin at least once during the past 5 years. The proportion of individuals who reported that they have ingested ivermectin every year during the past 5 years was 34.5% (95% CI: 30.8–38.4%), comparable between males (36.2%; 95% CI: 31.0–41.7%) and females (32.8%; 95% CI: 27.7–38.3%) (*χ*^2^ = 0.76, *df* = 1, *P =* 0.3833). A significant increase in the trend of compliance with ivermectin treatment was observed between the age groups (*χ*^2^ = 64.08, *df* = 3, *P <* 0.0001); 22.6% (95% CI: 16.3–30.4%) of participants aged 10–19 years (individuals < 10 years-old excluded), 20.4% (95% CI: 13.5–29.7%) of participants aged 20–34 years, 44.0% (95% CI: 35.1–53.4%) of participants aged 35–49 years, and 60.3% (95% CI: 53.1–67.1%) of participants aged ≥ 50 years declared having taken ivermectin tablets during the past 5 years.

The proportion of systematic non-compliers, that is those individuals who never ingested ivermectin tablets during the past 5 years, was 23.3% (95% CI: 19.9–27.1%), slightly higher in females (26.3%; 95% CI: 21.3–31.9%) than in males (20.4%; 95% CI: 15.9–25.7%) although statistically non-significant (*χ*^2^ = 2.5, *df* = 1, *P =* 0.1138). However, a significantly higher proportion of non-compliance with ivermectin treatment was observed between the age groups (*χ*^2^ = 24.59, *df* = 3, *P <* 0.0001). Participants aged < 34 years exhibited a higher proportion of non-compliance with ivermectin treatment compared to their older counterparts (*χ*^2^ = 64.08, *df* = 3, *P <* 0.0001). Overall, 31.6% (95% CI: 24.3–39.9%) of participants aged 10–19 years (participants < 10 years-old excluded), 36.6% (95% CI: 27.5–46.7%) of participants aged 20–34 years, 15.6% (95% CI: 10.0–23.6%) of participants aged 35–49 years-old, and 15.2% (95% CI: 10.7–21.1%) of participants aged ≥ 50 years declared that they have never swallowed ivermectin tablets during the past 5 years.

### Thirty-year trend of onchocerciasis in the Boutourou Health Area

**Fig. 2:**
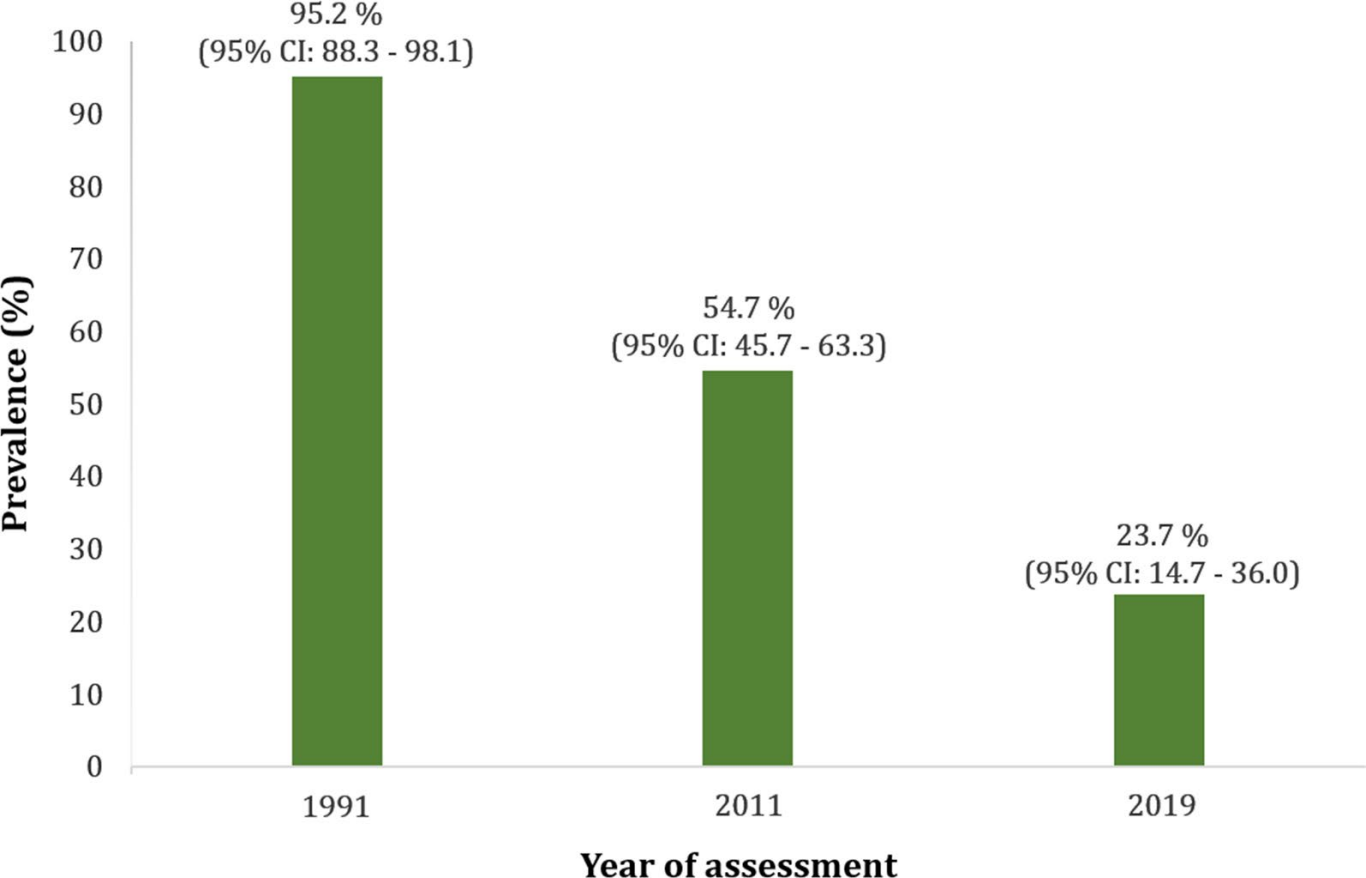
Trends in *Onchocerca volvulus* infection rates between 1991 (baseline) and 2019 (follow-up) in the Ndikinimeki Health District. Both the baseline (1991) and the follow-up (2011) surveys were carried out by the National Onchocerciasis Control Programme (NOCP), with the support of the African Programme for Onchocerciasis Control (APOC) (NOCP, unpublished reports)

A significant decrease in the prevalence of onchocerciasis was observed, between the present findings (2019) and both baseline data (1991) (*χ*^2^ = 41.59, *df* = 1, *P* < 0.0001), and the first decade trend (2011) (*χ*^2^ = 15.25, *df* = 1, *P* < 0.0001) (Fig. 2).

## Discussion

Based on the prevalence of microfilaridermia, onchocerciasis endemic levels have been classified as hypo-endemic (prevalence ˂ 35%), meso-endemic (35% ≤ prevalence ˂ 60%) or hyper-endemic (prevalence ≥ 60%) [26]. After two decades of CDTI, the Ndikinimeki Health District moved from hyper-endemic (prevalence = 95.2%) to hypo-endemic for onchocerciasis (prevalence = 7.0%). The intensity of infection, expressed either by the microfilarial densities (at the individual level) or by the CMFL (at the community level), was below 2 mf/ss. Finally, onchocercal clinical signs were almost inexistent in communities visited. These observations suggest that the CDTI that has been going on in the Ndikinimeki Health District for almost two decades has drastically reduced the burden of the disease, as observed in previous studies in Cameroon [27].

The higher proportion of infection among farmers and males could be explained by the occupational exposure and susceptibility of individuals. Farmers and males are more involved in outdoor activities, exposing themselves to infective blackfly bites. This agrees with the findings of Kamga et al. [16], who reported a higher prevalence of onchocerciasis in males than in females in the Yabassi Health District (Littoral Region, Cameroon). Furthermore, the high prevalence of the disease observed among participants aged 20–34 years may be due to the fact that individuals within this age group are usually the most active members in the communities and may have higher risk of getting infected because of their involvement in outdoor activities such as farming, sand mining and fishing. Indeed, a significantly higher prevalence of the disease was observed among farmers, compared to students, traders, housekeepers, and civil servants (nurses and teachers). In addition, because of their outdoor activities, these individuals are usually absent during household-based direct observance mass administration of ivermectin and this might therefore explain why these groups exhibit a low adherence to ivermectin treatment. Our observations disagree with the findings of Ikpo et al. [28] who reported a peak prevalence of onchocerciasis among individuals aged 41–50 years-old in the Oji River Local Government Area (Enugu State, Nigeria). In fact, onchocerciasis is known to be naturally cumulative, but treatments administered for 18–21 years might have disrupted this relationship, thus favoring the shift in prevalence peak among individuals highly exposed and poorly compliant with ivermectin mass treatments.

Despite the fact that preventive chemotherapies contributed to reducing onchocerciasis to hypo-endemic levels (microfilaridermia prevalence ˂ 35%) in all the surveyed Health Areas and communities, values were still above the expected level (≤ 20% for a pretreatment endemicity ≥ 80% assuming a therapeutic coverage of 65% [29, 30] in the Kiboum 1 and Kiboum 2 communities (Boutourou Health Area). This hotspot of onchocerciasis transmission may be due to the close proximity of these clusters to fast flowing rivers where blackflies breed. This is supportive of a change in the current strategies if one expects transmission interruption of onchocerciasis. Indeed, alternative treatment strategies (biannual CDTI, vector control using ground larviciding, vector control using vegetation slashing and clearing of blackfly breeding sites, test and treatment with the long-regimen macrofilaricidal drug doxycycline) have been developed by the African Programme for Onchocerciasis Control (APOC), and recently reshaped for adapted implementation purpose [31]. In the current situation with the persistence of onchocerciasis in a restricted hotspot area, implementation of multiple annual rounds of CDTI complemented with localized vector control by using for example ground larviciding might help boost the momentum towards the elimination of onchocerciasis in the Ndikinimeki Health District, and serve as a proof of concept for strategy improvement for countrywide elimination of onchocerciasis.

## Conclusions

This study reveals that the Ndikinimeki Health District is hypo-endemic for onchocerciasis after about two decades of CDTI. Despite the low prevalence and intensity of the disease in the study area, hotspots for onchocerciasis transmission was found in the Boutourou Health Area (around Kiboum 1 and Kiboum 2 communities) where alternative treatment strategies might be useful to prompt the elimination of onchocerciasis.

## Supplementary information

**Additional file 1: Table S1.** Prevalence and intensity of onchocerciasis in the different clusters of the Ndikinimeki Health District.

## Data Availability

All data generated or analyzed during this study are included in this published article and its additional file.

## Abbreviations

CDTI: community-directed treatment with ivermectin
CI: confidence interval
CMFL: community microfilarial load
mf/ss: microfilariae per skin snip
OR: odds ratio
PC: preventive chemotherapy
SD: standard deviation
