# Supplementary material for: Point prevalence mapping reveals hotspot for onchocerciasis transmission in the Ndikinimeki Health District, Centre Region, Cameroon
Source: Parasit Vectors. 2020 Oct 16;13:519. doi: 10.1186/s13071-020-04387-6 (PMC7565768; doi:10.1186/s13071-020-04387-6)
Supplement: Supplementary file 1 — Additional file 1: Table S1. Prevalence and intensity of onchocerciasis in the different clusters of the Ndikinimeki Health District. [file 13071_2020_4387_MOESM1_ESM.docx]

**Additional file 1: Table S1.** Prevalence and intensity of onchocerciasis in the different clusters of the Ndikinimeki Health District

| Cluster | Geographical Coordinates | No. individuals  examined | % prevalence  (95% CI) | Mean microfilarial  Density (mf/ss) (SD) | CMFL  (mf/ss) |
| --- | --- | --- | --- | --- | --- |
| Bonaberi | 4.75896 °N  10.83158 °E | 51 | 2.0 (0.0 – 10.4) | 0.108 (0.7702) | 0.060 |
| Kiboum 1 | 4.81962 °N  10.97827 °E | 57 | 19.3 (10.0 –31.9) | 1.395 (8.4143) | 0.383 |
| Kiboum 2 | 4.83849 °N  10.98296 °E | 59 | 23.7 (13.6 –36.6) | 1.814 (5.1986) | 0.538 |
| Makénéné Centre | 4.88640 °N  10.80601 °E | 51 | 3.9 (0.5 – 13.5) | 0.020 (0.0980) | 0.023 |
| Neboya | 4.62290 °E  10.85889 °N | 40 | 0.0 (0.0 – 8.8) | 0.000 (0.0000) | 0.000 |
| Ndikitole | 4.79334 °N  10.82353 °E | 61 | 3.3 (0.4 – 11.3) | 0.311 (1.7658) | 0.204 |
| New Town | 4.76980 °N  10.83941 °E | 58 | 5.2 (1.1 – 14.4) | 0.103 (0.4753) | 0.082 |
| Ndikoti 2 | 4.73287 °N  10.82794 °E | 28 | 3.6 (0.0 – 18.3) | 0.250 (1.3229) | 0.083 |
| Makenene Town Water | 4.88514 °N  10.80397 °E | 47 | 8.5 (2.3 – 20.4) | 0.979 (4.2643) | 0.289 |
| Hospital | 4.88422 °N  10.79645 °E | 62 | 3.2 (0.4 – 11.2) | 0.895 (5.2488) | 0.195 |
| Nyokon 3 | 4.92849 °N  10.75443 °E | 52 | 1.9 (0.0 – 10.3) | 0.019 (0.1387) | 0.021 |
| Ndokononoho | 4.64680 °N  10.78242 °E | 37 | 2.7 (0.0 – 14.2) | 0.230 (1.3974) | 0.084 |

*No.: number of; mf/ss: microfilariae/skin snip; CI: confidence interval; SD: standard deviation; CMFL: community microfilarial load*
